# Community Asset Mapping: Promoting Inclusion and Equity and Countering Stigma in Applied Substance Use Research

**DOI:** 10.3390/ijerph22101498

**Published:** 2025-09-28

**Authors:** William McGovern, Lydia Shrimpton, Hayley Alderson, Kim Hall, Monique Lhussier, Zeibeda Sattar, Paul Watson, Ruth McGovern

**Affiliations:** 1 School of Communities and Wellbeing, Faculty of Health and Wellbeing, Northumbria University, Coach Lane, Newcastle upon Tyne NE7 7XA, UK; kim.hall@northumbria.ac.uk (K.H.); monique.lhussier@northumbria.ac.uk (M.L.); zeb.sattar@northumbria.ac.uk (Z.S.); 2 Faculty of Medical Sciences, Population Health Sciences Institute, Newcastle University, Richardson Road, Newcastle upon Tyne NE2 4AX, UK; hayley.alderson@newcastle.ac.uk (H.A.); r.mcgovern@newcastle.ac.uk (R.M.); 3 School of Health and Nursing Science, Faculty of Health and Wellbeing, Northumbria University, Coach Lane, Newcastle upon Tyne NE7 7XA, UK; paul5.watson@northumbria.ac.uk

**Keywords:** stigma, health services, lived experience, co-production, qualitative research, equity, PWUS

## Abstract

People Who Use Substances (PWUS) are among the most stigmatised groups in society. Stigma associated with substance use is known to be detrimental to the individual’s wellbeing, and substance use is often used as a mechanism by policy makers and services to legitimise exclusion. PWUS often do not benefit from the drug and alcohol services that are available to them. Community Asset Mapping (CAM) is a strengths-based approach involving the re-engagement of communities through active involvement in research. There are criticisms and concerns about equity and the burden on participants involved in CAM projects; however, the broad aim of CAM is to identify and document the strengths and pre-existing resources that exist within a community. In the following study, we utilised CAM processes and principles in a large city in the Northeast of England to enable people with lived experience of substance use and practitioners working in drug treatment services to come together and identify resources in the form of services and groups that support recovery. In the process, we were concerned with identifying, engaging with, and involving groups that were known to the recovery community but also were not part of an existing recovery network. Qualitative data were obtained from semi-structured interviews (n = 13) and focus groups (n = 2). A reflexive thematic analysis approach was used to analyse the transcriptions, and from this we generated four themes: (1) community visibility, (2) ownership of the recovery agenda, (3) the impact of stigma and shame, and (4) the benefits of involvement. Our findings revealed a partly fragmented but also well-established, connectedand resourced local recovery community in the city. We were also able to identify a number of recovery groups and services that had previously not been known to the existing recovery community. Additionally, we identified that public and societal substance-related stigma continued to be a barrier that inhibited individuals and recovery groups from making themselves more visible and available to others.

## 1. Introduction

Stigma is an avoidable harm and yet still a key determinant of exclusion. It is associated with the maintenance of various forms of social and health-related harms and structural inequality [1]. At the broadest point, stigma is theorised as a process of disqualifying and marginalising individuals or groups based on negative perceptions of their characteristics, motivations, acts, attributes, and/or behaviours [2]. Stigma is widely accepted to be a harmful act and can be a social determinant of an individual’s health, as well as their sense of inclusion and value [3]. People Who Use Substances (PWUS) are among the most stigmatised groups in modern and westernised society, and a multitude of studies and reviews have identified that PWUS are known to experience stigma in a multitude of forms, be these institutional, societal, public, and/or private [4,5]. Stigma is complex and multifaceted, and public stigma refers to the negative attitudes and beliefs that society holds towards groups or individuals: it manifests as a source of harm when people respond negatively to others (prejudice), and then treat them differently (discrimination) or exclude them because of the status/identity/behaviours [5,6]. Private stigma usually occurs because of public stigma, but this form is associated with the individual developing negative beliefs about themselves and their attributes and then responding negatively to themselves (self-prejudice), excluding themselves, or denying themselves opportunities because of their perceptions of their own worth [6]. Like all forms of stigma, public and private stigma are harmful to experiences. The stigmatisation of PWUS is also described as being weaponized by those with power, such as policy makers, to justify exclusion from society [1]. This, in turn, creates barriers for PWUS to engage with and benefit from health and social care services, and hinders opportunities for involvement in supporting the development of health and substance-specific treatment and recovery services [4].

Collaborating and co-producing research with PWUS and involving them in research and the development of public services offer the potential to reduce stigma and inequality, which is associated with a range of social, societal, and personal benefits [7]. PWUS are known to be aware of stigma and inequality, and centring their perspective and lived experiences in co-produced research provides a lived experience and unique perspective from them about what best serves them [8,9]. Co-produced research encourages inclusivity in practice and benefits the individual by combatting stigma through empowerment and by providing a sense of worth and agency [8]. In addition, it has been identified that meaningful and authentic collaborations between People With Lived Experience (PWLE) and other key stakeholders can enhance the quality and relevance of strategies designed to reduce inequality and promote the uptake of public services [10]. However, despite the benefits associated with co-produced research [11], the lived experience of minoritised groups, such as PWUS, has often been under-represented in research design and development [12].

Community Asset Mapping (CAM) is associated with a wide range of methodological approaches and is a form of research and community development that is conducted collaboratively with communities of location, status, identity, interest, and experience [13]. All communities possess strengths and a range of assets within them; these resources can include services, but they can also include key individuals who are active in the community and who have skills, knowledge, social networks, and relationships. CAM is often represented in community research and accepted as a strengths-based approach, as well as a useful tool/method for assessing health-related needs, identifying resources, disparities, and inequalities within communities [13,14]. It is considered a more rigorous approach to include marginalised communities and voices in the scope, addressing and attending to gaps within service provision [14]. In our research, however, we also believed that conducting this research using CAM would enable us to begin bringing together PWUS, health and social care providers, and public health partners from a large city in England to identify resources in the form of key individuals and recovery services that supported the recovery (physical, psychological wellbeing, and social support/involvement) within the community. Recovery from substance use is a complex and contested concern; however, there is general agreement that recovery is an incremental process of change and involves improvements to an individual’s social position and psychological and social wellbeing [15]. To recover, or move towards recovery, individuals may need different types of resources or recovery capitals: recovery capitals (personal, social, community, cultural) are the internal and external resources that people can draw on to support their recovery [16]. The city in question already had an identifiable and existing “recovery community”, which was made up of independent recovery groups, nationally and internationally affiliated groups, community groups, and key independent individuals that met regularly and worked together to promote recovery and recovery services. This research was concerned with working in partnership with the existing forum and then using CAM to uncover and identify the existence of previously unknown or little-known (to the existing network) independently run recovery groups, self-organised groups, community groups, and key individuals that also supported recovery. The existing network was also concerned with exploring and understanding the opportunities these other groups offered their own members, and with exploring the potential contribution they could make in relation to furthering the inclusion and wellbeing of the wider community. The utilisation of the CAM approach allowed existing and known community members (those conducting CAM) to understand more about the actual and potential resources available to them, whilst also creating a context where they felt like they were making an active contribution to their own community [14]. The utilisation of the CAM approach is useful for identifying the ways in which barriers to participation, like stigma, can inhibit, restrict, and act against individuals and recovery groups in a way that makes these groups more reluctant to become visible and therefore available to others [17]. Overall, CAM is an inclusive and equitable way of working with communities, and more needs to be achieved in research and practice to enable opportunities for existing recovery communities to take action to change or improve their position and the overall psycho/social wellbeing of their members [17]. The discussion in this paper is concerned with exploring the relative merits of CAM as a more inclusive, equitable method of involvement, and we consider this to be an anti-stigma approach. Overall, this paper demonstrates the importance of collaborative working and methods, such as CAM, within the community, between commissioners, practitioners, and those with lived experience, to reduce stigma and promote inclusion and recovery. We conclude this paper by arguing for more inclusive and equitable methods like CAM to be used in research with PWUS and other marginalised groups, who are affected by inequality, thereby providing these groups with the opportunity to be involved in the development of health and social care services.

## 2. Materials and Methods

### 2.1. Research Design

This research was conceptualised, designed, and co-produced in partnership with the city’s existing recovery community group and funded by the city’s local authority Public Health team in the form of a “Community Capacity Building Grant”. This was a qualitative study, and a CAM approach was utilised because of the wealth of benefits associated with it [17]. This approach was also chosen since both the research team and the existing recovery community group were collectively concerned with identifying resources in the form of key individuals and lesser-known and less-involved groups/services. During the research, we utilised a range of involvement, engagement, and co-production techniques and strategies: importantly we recognised that there is no single formula method for co-production, and to assert one would be “counter to the innovation and flexibility that is implicit in co-produced research” [18] Engagement occurred as we engaged with and informed the forum and public members about our research and our starting aims and objectives. Engagement strategies were used to involve the public members we recruited more intensively in the research design [19,20,21], and implementation (identifying participants) and co-production occurred when public influence and opinions were used to design and produce topic guides, interpret data, and present findings during dissemination (such as co-presenters at conferences) of our research. A sub-group of the existing recovery community group members was formed and then involved in four themed and structured co-production sessions throughout the duration of the research: (1) reflecting on recovery needs and identifying services important to recovery, (2) mapping personal recovery journeys, (3) categorising the mapped services identified during CAM, and (4) authenticating interim findings from interviews with practitioners and service providers.

All of the recovery community sub-group members were given an information sheet detailing the study and an opportunity to ask questions before being given a public involvement consent form to sign. Each member of this group was remunerated using the National Institute for Health Research guidance for their involvement in each session [22]. Each individual structured session was designed to complement the next, and the principles surrounding our co-production approach were concerned with “valuing and nurturing” relationships with participants and then “supporting and enabling them” to draw on their potential in carrying out their roles and responsibilities in the research [18]. We continued our co-production work by asking participants to voluntarily recount and talk about their own experiences of recovery and engagement with recovery groups and services. We built on this in the next two sessions and invited participants to go out to their extended networks and speak about the perspectives of others in recovery whom they either knew personally, were involved with, or supported. We then worked with participants and began the process of co-producing a map of services from the previous workshops that they themselves and their peers had used to support their recovery. During these workshops, decision making was shared among researchers and participants, and steps were taken to make sure that everyone was involved in key decisions on research design, implementation, focus, analysis, and dissemination [18]. Multiple methods, including focus groups (n = 2) and semi-structured interviews (n = 13), were also utilised to gather data and to create a greater depth of understanding [19,20,21]. Following completion of the structured (workshops) process of involvement, members of the research team, LS/WM, attended monthly forum meetings to continue updating the members and to authenticate data [21] as they were gathered. Ethical approval was obtained for the study from the lead authors’ Ethics Review Institute Committee (4770) in 2023.

### 2.2. Sampling and Recruitment

To implement our CAM research design, we first needed to form a “recovery community sub-group” made up of people who were part of the established recovery network and who had insight into existing recovery services. We recruited six members of the existing recovery community group by giving a formal presentation of our research plans and opportunities for participant involvement. A diversity of characteristics, including past and present substance use, familial substance use, and consumption patterns, was sought for inclusion in the recovery sub-group. To be eligible to be a member of the sub-group, participants had to have experience of accessing or supporting someone in accessing recovery services and groups in the community, to be able to discuss their perceptions or involvement with the recovery community, and be over 18 years of age to be involved. All (n = 6) forum members who expressed an interest were invited to become part of the sub-group. To progress the research, we asked the sub-group members to identify key individuals whom they considered to be influential in their recovery and the recovery community. We realised our approach to sampling here ran the risk of recruiting and overrepresenting already well-connected individuals and groups [22,23,24]. With these potential criticisms and limitations in mind, the research team then approached these nominated individuals, provided an information leaflet and verbal explanation of the study, and invited them to participate. Those who agreed to be involved were invited to participate in a focus group and were also asked to identify other individuals whom they saw as key members of the recovery community. This combination of purposive and snowball sampling [25,26] resulted in a combination of individuals who were already part of the existing recovery network and those who were not. This included commissioned services and formal groups, as well as informal support networks organised by people who had previously used substances and were offering support (such as sponsorship) to others at earlier stages of recovery.

During the focus groups, members of the existing recovery network and key individuals who were outside of it were approached and recruited to take part in semi-structured interviews. At each stage of the research implementation, i.e., sub-group workshops, focus groups, and interviews, activities followed one another in a sequential way, with each stage informing the next as data were analysed and themes refined. Theoretically, our overall CAM approach was rooted in constructivism [27], and because of this, we believed that knowledge was not something that was inert or simply waiting to be uncovered among participants, but rather it is constructed by individuals as they interact with others in the world they are interpreting [27]. In terms of research design, data collection, and analysis, this meant that we made a commitment from the outset to involve participants at all stages of the research process, to utilise methods that enabled participants to share their perspectives, and to conduct sense-checking and interpretation exercises during analysis and consultation events [28]. Overall, this approach enabled us to develop more productive relationships with participants and gain a deeper understanding of how participants constructed knowledge and made meaning of their experiences of using substances, accessing recovery services, and navigating their recovery journey. Our approach also enabled us to engage with and understand participants’ knowledge and their real-world experiences, whilst enabling us as a research team to ultimately draw themes to explore and conclusions from their experiences [29]. All of those who were approached to be involved in the study (sub-group members, focus group members, and interviewees) agreed to be involved, and no one was excluded, see Table 1 reseach participants. A consent form was provided for every participant to sign in advance of the interview, and a verbal debrief [17] (because of the sensitive nature of the topics discussed) was also given to the participants following the focus groups and interviews.

### 2.3. Data Collection

Interviews and focus groups were the main methods used for data collection, and these were conducted between February 2024 and September 2024 by author LS. Topics explored included the following: the visibility, promotion, collaboration, and development of a recovery community; the inclusivity of the recovery community; the individual, social, and cultural benefits associated with a recovery community; the structural and social barriers to developing a recovery community; and how services support recovery and the recovery community. Our final sample included a range of previously known and previously unknown or lesser-known individuals and groups (to the existing network), independently run recovery groups, affiliated 12-step self-help groups, independent self-organised groups, community groups attached to static services, and key individuals who also supported recovery. The interviews lasted approximately 25–45 min, and the focus groups lasted approximately 40–60 min. All data collection was supported by a flexible topic guide, which was informed by previous literature and discussions that took place in the recovery sub-group sessions. Interviews were conducted in person (n = 9) and online, via Microsoft Teams (n = 4), in accordance with participants’ availability and preference. Both focus groups were conducted in person. The interviews and focus groups were digitally audio-recorded and transcribed verbatim by our external transcription service. Participants were allocated a participant identifier, and transcriptions were cleansed of any identifiable data to uphold anonymity. Field notes were also taken by LS to support the transcriptions and analysis process.

### 2.4. Data Analysis

Within the study, a reflexive thematic analysis approach was used to analyse the interview and focus group transcriptions [29]. Transcriptions were closely examined, read, and re-read by members of the research team (WM and LS) to allow for differing and comparable interpretations of the data. Analysis was informed by an interpretivist paradigm [30], which meant that we were committed to understanding the meaning and interpretation of social experience from the perspective of the individuals being studied. This approach allowed the researchers to inductively analyse the data with the understanding that participants’ experiences are unique and shaped by their individual perspectives on working within the recovery community [30]. A pre-existing rapport established in drug and alcohol practice between the researcher and participants, prior to the research being conducted, enabled the participants to share more openly during interviews. Data interpretation, interim, and final data analysis were also shared with the recovery community sub-group and discussed openly during wider recovery community meetings.

The reflexive thematic analysis process followed within this study involved six stages: (1) data familiarisation, (2) code generation, (3) theme generation, (4) theme review, (5) theme definition, and (6) result production. Double-coding was utilised at the outset of the data analysis, and WM and LS each analysed a sample (n-2) of the same transcript to generate initial codes and emerging themes. Initial codes and data examples from these transcripts were cut and pasted from Microsoft Word documents onto an Excel sheet and then organised into an indicative set of emerging themes by discussion among LS and WM. From this point, LS led the data collection, extraction, and analysis, and then codes and themes were discussed and refined between LS and WM during data analysis and management meetings. Decisions were made to include and remove codes/themes through discussion and with the involvement of the forum and the wider research group via workshops and presentations. Participants were not permitted to read individual transcripts, and instead, they were presented with the key themes and codes as discussion points during forum research meetings. During these meetings, participants discussed the relevance of the work, findings, and data that were emerging from the interviews, and they were able to contribute to the refinement of ideas. Data themes and codes among them were therefore validated by the forum and also by the wider team (HA, PW, ML, KH, ZS, RM) during the consolidation and final report writing processes. All of the authors and forum members were in agreement about the themes, codes, and structuring of the data.

## 3. Findings

Following the analysis of the qualitative data, we generated four key themes:

(1) Community visibility: visible to self, visible to those in recovery, no visible form, having to find recovery, visibility and hope, the need for visibility;
(2) Ownership of the recovery agenda: key players, community connectors, word of mouth, personal and professional responsibility, bridging the gap, all working together;
(3) The impact of stigma and shame: personal and private stigma, secrecy, hiding in plain sight, carers and families, fear;
(4) The benefits of visible and practical recovery: recovery city, social good, identity, belonging, social and emotional support.

### 3.1. Community Visibility

This theme describes and is concerned with illustrating and discussing the extent to which the recovery community was perceived to be visible to primary health and social care providers, and those both in/not in recovery.

During the sub-group meetings, focus groups, and interviews, participants were able to identify and describe a range of key individuals and organisations that support recovery. The groups identified included recovery groups that were affiliated with larger 12-step fellowships such as Narcotics Anonymous and Alcoholics Anonymous (NA and AA), as well as recovery groups that were attached to specific services, independent recovery groups run by their own members, and recovery groups supporting specific groups like women and minoritised communities. As we engaged with the recovery community that was known, it became immediately apparent that there were many different types of recovery organisations that worked specifically with drug and alcohol users and many others that sought to support the physical, social, and psychological wellbeing of PWLE of substance use. From our mapping process, it became apparent that everyone knew someone or an organisation; however, some groups were more well-known than others, and some were known but only to their members and not to the wider recovery community. Consensus among participants was that there was a well-established, expansive, and thriving range of recovery community groups/services in the city:

*“I’d say [the city] does have a really abundant recovery community […] there are lots going on”.*

(Participant 4, Service Provider of Recovery Groups, PWLE)

*“There is such a broad recovery network […] there’s so many fellowships for all sorts of recovery aspects […] it is amazing how much is out there”*.

(Participant 10, Service Provider of Fellowship Group, PWLE)

Alongside the consensus that there was a large and existing network of recovery groups/services was the shared agreement that there was a lack of recovery community visibility to those not in recovery in the city and among professionals, the media, and the wider non-using community. It was also widely recognised that there were many different organisations and groups that supported the physical, social, and psychological wellbeing of people in recovery that were not known to the wider recovery community. Participants recognised and shared general agreement that the recovery community, services, and support became more visible once a person was in a recovery service or group. They also reported that finding a recovery group/service was often the most difficult or important ‘first part’ of the recovery journey, as suggested by the two quotations below.

*“Trying to find somewhere in the first place is probably the biggest challenge”.*

(Participant 10, Service Provider of Fellowship Group, PWLE)

*“When you are in recovery, pretty soon you discover there is a whole world, but it doesn’t have high visibility, by and large”.*

(Participant 14, Service Provider of Recovery Service)

During interviews, individual professionals who supported PWUS and PWUS indirectly spoke about the visibility of the recovery community when they discussed how they encountered or came across recovery groups/services by default, rather than being able to identify and access a clearly defined and visible recovery community within the city:

*“I would not have known about the drug and alcohol recovery community […] it was much bigger, I imagined there not being much […] I’ve never really known that many people who have used recovery services and that […] I didn’t know it was there”.*

(Participant 12, Service Provider of Recovery Service)

*“Nobody told me that, that was a thing that I could have accessed […] you know someone who had been in the system had to sort of tell them about it and direct them”.*

(Participant 16, Service Provider of Recovery Group, PWLE)

Those who had been supporting (and often facilitating) recovery groups for a significant amount of time in the city were able to give a historical perspective of the recovery community’s growth and development. During the CAM process, these individuals were also able to ‘tell stories’ and reflect on how they felt the recovery community had grown organically and without structure in the city. These longer-term recovery community members also shared agreement with those newer to the city about the positive steps, like the well-attended recovery walk that had just occurred, a recovery conference that had been facilitated, and the formation of a city-wide Lived Experience Recovery organisation (LERO), which brought the separate parts of the recovery community and traditional drug and alcohol services together. Collectively, all participants in the study identified that more needed to be achieved to coordinate, structure, and raise the profile of the recovery community in the city. The benefits of this would be that recovery would be more visible and therefore less underground:

*“If is it not visible, it’s underground. And I think, a lot of people, when they come into recovery have lived in a world underground, whether it be like, criminal or shame or stigma or using behind curtains, or whatever, so we have got to make it visible”.*

(Participant 6, Service Provider of Recovery Groups, PWLE)

### 3.2. Ownership of the Recovery Agenda

This theme is concerned with exploring who participants perceived to be the key players and then who should be responsible for owning and promoting the recovery community and services in the city.

There was an agreement amongst research participants that people in recovery, recovery services, and traditional drug and alcohol services all had an individual and shared role to play in the development and promotion of the recovery community. Among participants were traditional drug and alcohol treatment providers who spoke at length about taking responsibility and ownership of recovery by supporting the development of groups within formally commissioned services, facilitating the involvement of groups, and allowing groups/services to co-locate with them:

*“Because we’re both based here, we’ve made a really good connection […] I think it is definitely improving. There definitely has been a change and we are much more connected as a drug and alcohol service community, and I think…the barriers are coming down and we’re networking better and we’re having better relationships and getting to know people more”.*

(Participant 13, Practitioner of Family and Carer Service)

However, formally commissioned drug and alcohol services also reported that there were barriers, such as fiscal short-term funding, lack of operational caseloads and time, and staff turnover that limited their scope and ability to take responsibility and ownership within recovery communities. During interviews, these participants and others spoke at length about the ways in which a lack of funding limited the provision of recovery services. In particular, it was highlighted that the needs of some groups were not adequately met; for example, the ability of recovery services to respond to the needs of parents was limited by a lack of childcare provision for parents attending groups and services.

*“If you don’t have childcare, you can’t access things that are not suitable for children. This is a big drop off and that’s why you see relapse because [they’re] feeling isolated in that way. Or even if there was community spaces where recovering parents could come together with their peers and children. I think that is an important area as well”.*

(Participant 4, Service Provider of Recovery Groups, PWLE)

Traditional drug and alcohol providers in the study were very keen to praise the recovery community in its breadth and scope, and also shared an enthusiasm for taking ownership of their own practice and being more open to the idea of supporting recovery groups. In the process, however, practitioners and organisations spoke about their confusion and lack of understanding about the different approaches, philosophies, and processes for engaging people in recovery services. Taking ownership and responsibility for promoting recovery groups was made difficult because of the lack of time and resources available, but also because of a lack of knowledge and understanding practitioners and services had:

*“I think the split comes […] from a lack of information, lack of knowledge [support available] given to services. And that comes not just the recovery model [philosophy/approach] giving information to other people……it is also the service [recovery groups] not linking in with each other”.*

(Participant 11, Outreach Practitioner, PWLE)

Members of the existing recovery organisations and representatives of the recovery community themselves recognised that they could take further ownership in relation to networking, being visible, and “linking” with other recovery organisations and more traditional forms of drug and alcohol services. Representatives of recovery groups who were not part of the recovery network identified that they took ownership and promoted their own service to community members and engaged with other recovery organisations on collective concerns, like activism and celebration events. Like the practitioner above, however, these groups, which were not networked into the wider recovery network, also recognised that the recovery community at times took a siloed approach and did not wholly promote other recovery organisations and groups. Whilst there were recovery organisations that supported other recovery groups in the study, it was also accepted that some recovery groups focused on their members and their group and as a result did not actively promote other groups to their members. Those participants who had an awareness and understanding of different recovery groups were able to discuss these concerns during an interview:

*“At the minute, I feel it is a little disjointed […] what I find is that the groups don’t mix; so [name] and [name] don’t mix, [name] and [name] don’t mix either”.*

(Participant 6, Service Provider of Recovery Groups, PWLE)

### 3.3. The Impact of Stigma and Shame

This theme is concerned with the perceptions and experiences of stigma as limiting factors in the development of the recovery community/support, as well as people’s engagement with it.

Stigma was discussed as a complex concern, and various forms of stigma (healthcare professional, public, private, self, societal, and label avoidance) were described by participants as a barrier to the ways in which people engaged with the recovery community and how the recovery community functioned. Despite participants with lived experience of substance use identifying that they were no longer using substances and were “in recovery”, they spoke about public stigma and how they found it difficult to be involved with the recovery community because they felt they were still judged by society for who they were previously. Public stigma occurs when people have negative beliefs about individuals and groups in society, and they then have negative emotional reactions to them (prejudice) and treat them wrongly (discrimination) or badly because of this [7].

*“You talk about your heroin addicts, and you always see a little back alley somewhere, injecting, or in a horrible bedsit, dark dingy, all of the things that are portrayed in the media and in your soaps […] A “normal” [our emphasis] person living their life, they might not know about recovery, I guess all they see is the media and how addiction is portrayed rather than how recovery is portrayed.”*

(Participant 8, Service Provider of Recovery Service)

Stigma did not only affect the individual using substances or not using substances, as was the case with participants. A small number of participants also spoke about the impact of stigma on themselves, including both public and private stigma, and also about the impact of associative stigma and the effect this had on families. Associative stigma occurs for people who care for or are associated with people who use/have used substances, and here it refers to the negative perceptions and discrimination that are directed towards individuals who are close to someone or who care for someone experiencing stigma [4]. Associative stigma can make it difficult for people to engage with recovery services, as well as for other non-using family members to engage with services and support their own carers and wider recovery community.

*“I think there’s potentially an element of hidden population of people, and [that’s] families. So, families that are affected […] there’s a whole other bunch of people there who equally have been maybe traumatised […] So, I think there could be more scope done on that side of things, in terms of like, recovery.”*

(Participant 18, Practitioner of Recovery Service)

There was also a perception among participants that access to recovery services and opportunities to support their own wellbeing was something that had to be negotiated in secrecy because of public and private stigma. Stigma processes and anticipation of stigma were very much normalised and discussed as an everyday part of the journey for those seeking recovery. By and large, it resulted in people in recovery hiding their recovery status from others in society and from those outside their own network. These factors meant that people in recovery are often, but certainly not always, hidden in plain sight, and as a result of stigma, they do not make themselves visible because of the psychological and social harms that would come from their status (and previous status) being known to others.

*“Addiction has always felt like something that is full of shame and is a secret […] we don’t admit that […] and we don’t tell people that we’re going to a [name] meeting.”*

(Participant 16, Service Provider of Recovery Group, PWLE)

During focus groups and interviews, participants spoke at length about stigma and shame, and they also spoke about the ways in which self-stigma could affect an individual’s self-worth and self-belief in relation to their ability to make an active contribution to their own lives and the lives of others as someone in recovery.

*“People who are ashamed, they’re embarrassed. They blame themselves and it is actually lack of education on other people’s behalf, because you know what, nobody woke up, saying “I want to be an alcoholic.” Of course they didn’t. Or “I want to be a drug addict.”*

(Participant 13, Practitioner of Family and Carer Service)

*“I do think that’s one of the big issues, is self-stigma. It’s like believing what you think other people think of you holds a lot of people back”.*

(Participant 5, Outreach Practitioner, PWLE)

Experiences of combatting and countering the impact of self-stigma by developing self-belief (see the next theme also), making an active contribution to one’s own wellbeing, and making a positive contribution to one’s recovery community were discussed at length by PWLE during the study. Self-stigma occurs when an individual who is stigmatised develops negative self-perceptions about themselves and their own character, and then has a negative emotional reaction to themselves. This can leave the stigmatised person with low self-worth and negative beliefs about their own self-efficacy and abilities [26]. It was also apparent that some recovery sub-groups, like mothers with children, had to negotiate and consider several complex concerns and decisions, which included stigma, shame, and fear of accessing recovery services, groups, and support.

*“It’s not even just stigma, it’s stigma and fear, so if you’re a mum and you’ve got children and you’re struggling with addiction, what’s going to happen if you ask for help? It’s going to get much worse. It’s not going to get better. You’re told: Be honest and ask for help and it’ll get better. It doesn’t always happen like that. It doesn’t work like that at all; sometimes you can get your children removed. You know, it’s quite scary.”*

(Participant 4, Service Provider of Recovery Groups, PWLE)

### 3.4. The Benefits of Visible and Practical Recovery

This theme builds on the lack of visibility and the tensions around ownership of the recovery agenda raised in previous themes. It describes and is concerned with exploring participants’ perceptions of the benefits of having a functioning and visible recovery community.

Each participant in this study with a lived experience of substance use had a highly individualised story to tell as part of their recovery journey. These participants celebrated and shared their stories with us during sub-group workshops, focus groups, and interviews. Without exception, each of these participants also identified that having a visible and open recovery community increased the likelihood that people would come forward, identify with others, and then engage with others to support their recovery-oriented and wellbeing needs. During interviews, participants also discussed how difficult it was initially to bring themselves to ask for help from others because of their low self-worth, and also because they were isolated and did not have access to wider social networks. They also spoke about how having a visible recovery community was beneficial and countered some of these concerns:

*“It can feel you don’t want to admit something, ‘cos it can naturally feel isolating, but if you know there’s a whole community that are visible, you don’t feel quite so scared about admitting you’ve got an issue, and the support is there.”*

(Participant 16, Service Provider of Recovery Group, PWLE)

Having a more visible recovery community was also seen as valuable by service providers. Practitioners in particular identified that having a visible and functioning recovery community was good for individuals accessing services, the city, and themselves in relation to their work in raising awareness of needs and providing services to improve individual and social wellbeing. Participants spoke at length about the importance of choice and autonomy when accessing support, and each recognised that there was not a ‘one-size-fits-all’ recovery service. Having a visible and open recovery community meant that PWUS were able to engage with existing recovery group users with a view to ultimately choosing a group or recovery service that they could identify with, and which met their own unique needs.

*“There are so many paths as well and everyone has their own path to choose. Often, it’s a game of experimentation. You try something. You know, if that doesn’t work for you, maybe that’s not right yet; we’ll try something else, see if that works.” […] “Having that variety of choice… You know, there may be some venues you don’t like going to, because of the part of town they’re in or because the building itself, you might have negative associations so there’s that aspect of variety and choice.”*

(Participant 10, Service Provider of Fellowship Group, PWLE)

Participants identified with others, and this provided them with social connections and motivations to become involved and to change. They also identified that having a visible community provided them with a sense of hope and that they felt empowered by being able to identify openly as someone in recovery. Not being stigmatised for this was important, as was being able to move fluidly to access multiple forms of recovery services. There was a broad recognition amongst participants that having the opportunity to identify and engage with many different forms of recovery groups was important. Some participants were able to identify a group, engage with a group, make a connection with a group, and then go on to become a member. Others spoke at length about different experiences of moving between groups to try and find a suitable program or model of self-help to follow and identify with. Those who managed to navigate different types of recovery groups before settling on a group spoke about the empowerment of being able to choose a group that aligned with their needs as part of the overall recovery process. Overall, participants recognised that raising the profile of the recovery community normalised their existence in society and started a process of countering the stigma and shame that are associated with PWUS and those who are seeking to better their lives and positions by improving their wellbeing and accessing recovery services and groups.

*“I think it just stops it also, even for people who don’t have addictions, it stops it being looked at as a negative. Because if you just see it visibly and positively… And then if they happen to know anybody, they can say, “oh, have you seen or heard about these?” So, it becomes a more accepted normal thing, rather than a hidden away […] When you’ve got a visible community and you feel like, actually, I wouldn’t mind being a part of that, you’re more willing to be open… Sober.”*

(Participant 16, Service Provider of Recovery Group, PWLE)

## 4. Discussion

Community Asset Mapping (CAM) is an inclusive, beneficial, and equitable form of research that can reduce public stigma and encourage marginalised groups, communities, and those who provide services to them to come together and consider their strengths, their positive attributes, and collaboratively build resources for their community [31,32]. By conducting this research using CAM, this project was able to support an existing network of recovery organisations and services to identify, engage with, and involve key individuals and recovery services that were not part of the existing recovery support network. Our findings, however, illustrated that individuals will actively exclude themselves to avoid public stigma associated with substance use and also that public and private stigma can increase as an individual moves towards bettering their wellbeing by engaging with a recovery-oriented service or group [33]. Like others, we have also found that different forms of public, self, and private stigma can have long-term and debilitating psycho/social effects on a person, their self-worth [34], and their beliefs about their attributes and abilities [35], and can also lead to inequality influencing the self-efficacy and willingness of individuals to engage with others and access services [36]. Our utilisation of the CAM approach to research design created a space for PWLE of stigmas to come together with professionals to tell positive stories about their experiences of accessing and engaging with recovery groups and counter stigma by explaining how they took steps to improve their own positions in relation to increased psycho/social wellbeing [12]. In the process, we were able to identify the importance and benefits of having a visible recovery community whilst also identifying the barriers and the ways in which concerns with stigma limited the visibility of groups to their community. In addition, our approach to research design supported PWLE to reflect on their positive attributes and “reauthor” their stories by focusing on factors like resilience, empowerment, and their abilities and strengths as they engaged with and navigated recovery services [37]. The storytelling we heard was curated in forum discussions and by individuals within interviews with us; we did not seek to edit these or curtail them; rather, we let them occur naturally and within the forum/interview context. Hence, whilst we did not specifically seek to assess or measure the individual therapeutic value and benefit of storytelling as an approach in this project, we did know from our own work and the work of others [38] that storytelling approaches are beneficial to the individual as they move the focus away from harmful and negative narratives of individuals to more empowering and adaptive ones that foster healing, positive self-growth, and increased feelings of individual wellbeing [15]. This is especially so when individuals can contribute to identifying resources for their community by telling stories and sharing narratives that start from positions of disadvantage and personal difficulty and end with successful, identifiable, and positive outcomes [5].

CAM approaches not only create space within research design and implementation for PWUS to share positive stories about their experiences and themselves [37], but they also allow participants to identify community resources (such as services and groups in our study) that have the potential to actively contribute or serve others [17]. At every stage of this study, participants were involved in identifying city-based support services that they and others had utilised as part of their recovery journey. Theoretically, the idea that individuals can benefit in relation to their own social and psychological wellbeing through helping others in their community is well discussed and explored [39,40,41]. The benefits identified in studies for the helper include an increased sense of empowerment and competence, as opposed to stigma [40,41,42], increased psychosocial wellbeing and self-efficacy [41], and further helping commitment, activism, and validation of a new (positive) identity [40,41]. It must be recognised that the benefits of helping are experienced and felt more strongly by the helper in one-to-one relationships, where the helper actually supports change in relation to a positive outcome [39]. However, even when the relationship between the helper and the person being helped is less direct, as it is in our study, the overall effect on the wellbeing of the helper across different relationships and contexts, such as family, friends, community members, and even strangers, can be moderate [43].

People who are endeavouring to better their position and overall wellbeing are often left out of stories about their own lives. The CAM approach is a useful approach for engaging professionals and bringing communities and organisations/practitioners together in more equitable, democratic, and transparent relationships with people who have used services or been stigmatised [44]. As a sustainable result, and after the dissemination of our findings, the forum we worked with and a group of professionals from the city in which the research was conducted came together and formed a Lived Experience Recovery Organisation (LERO). In a more critical context, it has been identified that those conducting CAM need to be aware of critical concerns with equity, inclusion, power, fairness, and representativeness of participants throughout the process [45]. However, CAM is also a highly persuasive strategy for creating a context for challenging the existence of any negative perceptions, attitudes, beliefs, behaviours, and the stigma that can exist among healthcare professionals and organisations [46]. By engaging with professionals who provided services and bringing them together with PWLE and the recovery community, we were able to use CAM to explore where these two different groups shared priorities, concerns, and how each perceived the other in relation to working together. In utilising this approach, we encouraged professionals to consider and listen to stories of people trying to find a foothold in recovery, and in the process, we encouraged them to understand the experiences of those seeking recovery and to be more empathic and positive in relation to considering the strengths of the groups they support, whilst considering how they, as professionals, benefit from involving them to support practice [13]. Whilst we share agreement and recognise that CAM is still a very novel approach to public health research [13], we also agree with theorists [47,48] who argue that CAM approaches that combine education about stigma and contact with individuals with lived experience of stigma will reduce incidences of prejudice, avoidance, and discrimination among professionals with responsibility for delivering patient-centred, quality care.

### Limitations

This study has some limitations. It was specific to the city in which it was conducted, with a small sample size in relation to the study population and limited diversity in terms of race, indigeneity, identity beyond the binary, and migration status, which shaped the experience of participants. It is also limited because the research was conducted in a single city, and its findings may be less applicable to other cities, locations, and settings. It could also be interpreted as being biased, as it only briefly acknowledges critical concerns with the CAM approach and predominantly considers and reports on the perceptions of the strengths of participants as they discussed their engagement with the recovery community. Theoretically speaking, the categories we utilised to define recovery services and groups were very broad, and this was partly informed by confidentiality and ethical concerns intended to protect identity. There will also be groups that we missed, but we would argue that this study has gone a long way towards raising and giving a voice to the lived experiences and perspectives of others. There is clearly a need for more research into the utilisation of CAM as an approach to research, and there is also a need to understand stigma from the perspectives of those who have experienced it, especially stories about the experiences and strategies of those who have been able to overcome it and move on.

## 5. Conclusions

Concerns with stigma-related prejudice, discrimination, and unfair treatment do not end when PWLE of substance use take action to change or improve their position and overall psycho/social wellbeing. In many individual cases, societal and public stigma very much stays the same for marginalised communities, and for many, private and public forms of stigma can actually increase and continue to fuel disparities in health, wellbeing, and health outcomes. CAM strengths-based approaches, whilst still considered a novel approach in Public Health research, provide real-life opportunities not only for reducing or countering stigma, but also for making services more informed and relevant. Anti-stigma considerations, approaches, and research that are considered via an anti-stigma lens offer the opportunity for more impactful and activist-led research.

## Figures and Tables

**Table 1:** Research participants.

Domain	Number of Participants	Gender	Role
CP Session 1	(n = 3)	3F	Sub-Group Members (n-1 carer)
CP Session 2	(n = 4)	3F 1M	Sub-Group Members (n-1 carer)
CP Session 3	(n = 27)	13F 14M	Sub-Group and Forum Members (n-3 carers)
CP Session 4	(n = 13)	8F 5M	Sub-Group and Forum Members (n-3 carers)
Focus Groups	(n = 11)	6F 5M	Professionals and PWLE from Recovery Groups
Interviews	(n = 13)	3F 10M	Professionals and PWLE from Recovery Groups

Note. CP: Co-Production.

## Data Availability

The raw data supporting the conclusions of this article will be made available by the authors on request.
